# Cocaine use is associated with cerebral white matter hyperintensities in HIV disease

**DOI:** 10.1002/acn3.51854

**Published:** 2023-07-20

**Authors:** Christina S. Meade, Ryan P. Bell, Sheri L. Towe, Christopher D. Lascola, Kareem Al‐Khalil, Matthew J. Gibson

**Affiliations:** ^1^ Department of Psychiatry and Behavioral Sciences Duke University School of Medicine Durham North Carolina 27710 USA; ^2^ Brain Imaging and Analysis Center Duke University Medical Center Durham North Carolina 27710 USA; ^3^ Department of Radiology Duke University School of Medicine Durham North Carolina 27710 USA

## Abstract

**Background:**

White matter hyperintensities (WMH), a marker of cerebral small vessel disease and predictor of cognitive decline, are observed at higher rates in persons with HIV (PWH). The use of cocaine, a potent central nervous system stimulant, is disproportionately common in PWH and may contribute to WMH.

**Methods:**

The sample included of 110 PWH on antiretroviral therapy. Fluid‐attenuated inversion recovery (FLAIR) and T1‐weighted anatomical MRI scans were collected, along with neuropsychological testing. FLAIR images were processed using the Lesion Segmentation Toolbox. A hierarchical regression model was run to investigate predictors of WMH burden [block 1: demographics; block 2: cerebrovascular disease (CVD) risk; block 3: lesion burden].

**Results:**

The sample was 20% female and 79% African American with a mean age of 45.37. All participants had persistent HIV viral suppression, and the median CD4^+^ T‐cell count was 750. Nearly a third (29%) currently used cocaine regularly, with an average of 23.75 (SD = 20.95) days in the past 90. In the hierarchical linear regression model, cocaine use was a significant predictor of WMH burden (β = .28). WMH burden was significantly correlated with poorer cognitive function (*r* = −0.27). Finally, higher WMH burden was significantly associated with increased serum concentrations of interferon‐γ‐inducible protein 10 (IP‐10) but lower concentrations of myeloperoxidase (MPO); however, these markers did not differ by COC status.

**Conclusions:**

WMH burden is associated with poorer cognitive performance in PWH. Cocaine use and CVD risk independently contribute to WMH, and addressing these conditions as part of HIV care may mitigate brain injury underlying neurocognitive impairment.

## Introduction

Neurocognitive impairment (NCI) remains a prevalent complication of HIV disease, even in patients with sustained HIV viral suppression, with estimated prevalence rates ranging from 15 to 55% across populations.^1^ In the era of combination antiretroviral therapies (cART), milder forms of HIV‐associated neurocognitive disorders predominate,^2^, ^3^, ^4^, ^5^, ^6^ but nevertheless have real‐world impacts on daily functioning and are predictive of increased morbidity and mortality.^7^, ^8^, ^9^, ^10^, ^11^, ^12^, ^13^ These cognitive deficits correlate with neuronal injury due to chronic HIV disease that occurs through numerous mechanisms, including persistent immune activation and low‐grade inflammation.^14^, ^15^, ^16^, ^17^ As persons with HIV (PWH) now have nearly average life expectancies,^18^ normal aging processes and comorbid conditions likely contribute to the development and expression of NCI.

White matter hyperintensities (WMH), which have been observed at higher rates in PWH,^19^, ^20^, ^21^, ^22^, ^23^ are associated with NCI.^24^, ^25^, ^26^ The histopathology of WMHs is heterogeneous, including myelin pallor, demyelination and axonal loss, and mild gliosis.^27^, ^28^ The majority of WMH result from chronic ischemia associated with cerebral small vessel disease, which can cause vascular hypertrophy and microvascular remodeling.^29^, ^30^, ^31^, ^32^ While the biological mechanisms leading to WMH are not fully understood, possible factors include chronic hypoperfusion, increased permeability of the blood–brain barrier, vascular endothelial dysfunction, and inflammatory responses.^33^ Studies have linked WMH to circulating levels of inflammatory markers, including tumor necrosis factor alpha (TNF‐α), interleukins (particularly IL‐6), interferon γ‐induced protein 10 (IP‐10, also called CXCL10), monocyte chemoattractant protein 1 (MCP1, also called CCL2), and myeloperoxidase (MPO).^34^, ^35^, ^36^, ^37^, ^38^

Aging and cardiovascular disease (CVD) risk factors such as hypertension, diabetes, and smoking are the primary predictors of WMH in the general population^39^, ^40^ and in PWH specifically.^20^, ^22^, ^41^, ^42^ It is believed that chronic immune activation and persistent low‐level inflammation associated with HIV disease may affect microcirculation and contribute to increased risk for WMH.^19^, ^43^, ^44^, ^45^ WMH in PWH may also reflect legacy effects of neuronal injury that occurred during untreated phases of disease. One study reported that longer time spent with a CD4^+^ T‐cell count below 500 cells/μL (a marker of immunosuppression) was predictive of total WMH load,^22^ although most studies have found no relationship between HIV disease characteristics and WMH.^20^, ^23^, ^42^, ^46^, ^47^ In PWH, WMH have been associated with worse global cognitive functioning, although the specific domains affected varied across studies.^22^, ^41^, ^42^

Cocaine use is disproportionately common in PWH and may contribute to neuronal injury. The most recent surveillance by the CDC of adults with HIV reported past year prevalence rates of 5% for non‐injection cocaine and 3% for crack cocaine.^48^ By comparison, the past‐year prevalence of use among US adults was 2% for any form of cocaine, including crack cocaine.^49^ Cocaine is a potent central nervous system stimulant that has been linked to increased risk of ischemic stroke and intracerebral hemorrhage.^50^, ^51^, ^52^ Acute cocaine exposure causes a sudden transient increase in blood pressure, which can rupture leaky and weak vessels. Other mechanisms for cocaine‐induced lesions include cerebral arterial vasoconstriction, atherosclerosis, vasculitis, disruption of blood–brain‐barrier integrity, and neuroinflammation.^53^, ^54^, ^55^ However, the contribution of cocaine on WMH burden in PWH has received little attention. One study found that a history of cocaine use disorder was not associated with increased risk for WMH load in PWH,^20^ although the recency and frequency of cocaine use were not reported.

WMH can be measured quantitatively using non‐invasive magnetic resonance imaging (MRI). Specifically, T2‐weighted fluid‐attenuated inversion recovery (FLAIR) imaging is the industry‐standard sequence for delineating WMHs. The total WMH volume is an important determinant of their clinical relevance.^56^, ^57^ The current study quantified WMH burden in a sample of PWH who were treated with cART and had sustained HIV suppression. Our specific aims were to (1) investigate the independent contribution of cocaine use to WMH, after accounting for age and common CVD risk factors, (2) examine the correlation of WMH volume to cognitive function, and (3) explore the possible association of peripheral biomarkers of immune activation and inflammation to WMH load. We hypothesized that PWH who used cocaine regularly would have a greater WMH burden, and that WMH burden would correlate negatively with global cognitive function. We further expected that an inflammatory cascade would mediate the relationship between cocaine use and WMH burden.

## Materials and Methods

The data in this report were collected as part of a larger project on neuroimaging and immunological features of NCI in PWH.^58^ The study was approved by the Duke Health Institutional Review Board, and all participants provided written, informed consent.

### Participants

PWH aged 18–59 years were recruited from infectious diseases clinics in the Raleigh/Durham area of North Carolina, USA. Participants met the following criteria: stable ART for >1 year, nadir CD4 cell count of ≤350 cells/mm^3^ or HIV infection of ≥5 years; and sustained viral suppression (plasma HIV RNA < 200 copies/mL) for >1 year. Exclusion criteria included perinatal HIV infection; <9 years of education; BMI > 40; English non‐fluency or illiteracy; severe learning disability; severe head trauma with loss of consciousness >30 min and persistent functional decline; serious neurological disorders not caused by HIV, including stroke; acute CNS infection or chronic CNS infection with residual symptoms; severe mental illness or acute psychiatric symptoms; systemic autoimmune disease; inpatient hospitalization within 30 days; use of an immunomodulatory medication, steroid, or antibiotic within 30 days; contraindications to MRI; and other underlying or uncontrolled medical illness with altered cognition.

The sample was stratified by cocaine history. A positive history (COC+) was defined by lifetime history of regular use and ≥12 days of use in the past 90 days or a positive urine screen, while a negative history (COC−) was defined as no regular use in the past 10 years, no use in the past 90 days, and a negative urine drug screen. Alcohol, cannabis, and nicotine use were allowed, and controlled substances such as benzodiazepines were permitted if prescribed. For other illicit drugs of abuse, participants were excluded for: a positive urine drug screen; >2 days of use within 30 days; >2 years of regular use within the past 20 years; and any regular use in the past 5 years.

### Substance use assessment

The Addiction Severity Index‐Lite (ASI‐L), a semi‐structured interview, was administered to characterize lifetime substance use and associated problems.^59^ Module E of the Structured Clinical Interview for DSM‐5 (SCID‐5) was used to assess substance use disorder symptoms.^60^ Frequency of use in the past 90 days for cocaine, marijuana, and other substances was facilitated with Timeline Follow‐Back methodology.^61^, ^62^ A nine‐panel urine toxicology screen that tested for cocaine, amphetamine, barbiturates, benzodiazepine, methamphetamine, opioids, methadone, marijuana, and oxycodone assessed recent drug use.

### Clinical data

Participants provided a release for the study team to obtain copies of their healthcare records. This information was used to confirm no exclusionary conditions, and to abstract relevant clinical data. HIV disease characteristics included date of diagnosis, nadir and current CD4^+^ T‐cell counts, current antiretroviral regimen, and most recent plasma HIV viral load. The following clinical diagnoses were coded as present or absent: diabetes, hypertension, hyperlipidemia, and any prior CVD such as coronary artery disease and cardiomyopathy. Body mass index (BMI) was calculated from weight and height measured at last clinical visit. Participants reported frequency of smoking in the past 3 months. As in prior studies,^21^, ^42^, ^63^ a composite CVD risk score was calculated as the sum of the following risk factors: diabetes, hyperlipidemia, hypertension, CVD diagnosis, obesity (BMI ≥ 30), and current smoking. Total scores ranged from 0 to 6.

### Neurocognitive function

Neuropsychological testing was administered by Bachelor's‐level staff member under the supervision of a licensed clinical psychologist. The 60‐min battery of tests spanned seven domains of cognitive function:

1. *Attention:* Paced Auditory Serial Addition Task‐50—total number correct^64^; WAIS‐IV Digit Span subtest—total number correct^65^; WAIS‐IV Letter‐Number Sequencing subtest—total number correct^65^
2. *Information Processing:* Trail Making Test Part A—number of seconds to completion^66^; Wechsler Adult Intelligence Scale‐IV (WAIS‐IV) Coding subtest—total number correct^65^; Stroop Color and Word Test color naming score—total number of items completed^67^
3. *Learning (immediate recall):* Hopkins Verbal Learning Test—Revised (HVLT‐R)—total number of words recalled on trials 1–3^68^; Brief Visuospatial Memory Test‐Revised (BVMT‐R)—total score for figures recalled on trials 1–3^69^
4. *Memory (delayed recall):* HVLT‐R—total number of words recalled on trial 4^68^; BVMT‐R—total score for figures recalled on trial 4^69^
5. *Executive function:* Stroop Color and Word Test interference score—difference between actual and predicted score on the Color‐Word trial^67^; Trail Making Test Part B—number of seconds to completion^66^; Wisconsin Card Sorting Test‐64 (WCST)—total errors^70^
6. *Verbal fluency:* FAS letter fluency—total number of words generated; and category fluency—total number of animals generated^71^
7. *Motor skills*: Grooved Pegboard Test dominant and non‐dominant hand—number of seconds to completion^72^

Using published normative data,^64^, ^65^, ^73^ raw scores for each test were converted to demographically corrected *T*‐scores (M = 50, SD = 10). Domain *T*‐scores were computed by averaging the *T*‐scores for the tests within each domain, and a global *T*‐score was computed by averaging the 7 domain *T*‐scores.

### MRI data acquisition and processing

All MRI scans were performed at Duke University Hospital using a 3.0T GE Discovery MR750 scanner with an 8‐channel head coil. High‐resolution T1‐weighted images were recorded using a spoiled echo sequence (voxel size = 1 mm^3^, repetition time [TR] = 8.16 ms, echo time [TE] = 3.18 msec, field of view [FOV] = 256 mm^2^, 12° flip angle, 168 interleaved slices). Fast‐spin echo T2‐weighted FLAIR images were acquired in oblique axial orientation of the full brain with the following parameters: voxel size = 0.4 mm^2^ × 5 mm, TR = 10000 msec, TE = 128 msec, TI = 2337 msec, FOV = 220 mm^2^, 111° flip angle, 27 slices. Total acquisition time for FLAIR was 3:30 min.

**Figure 1:**
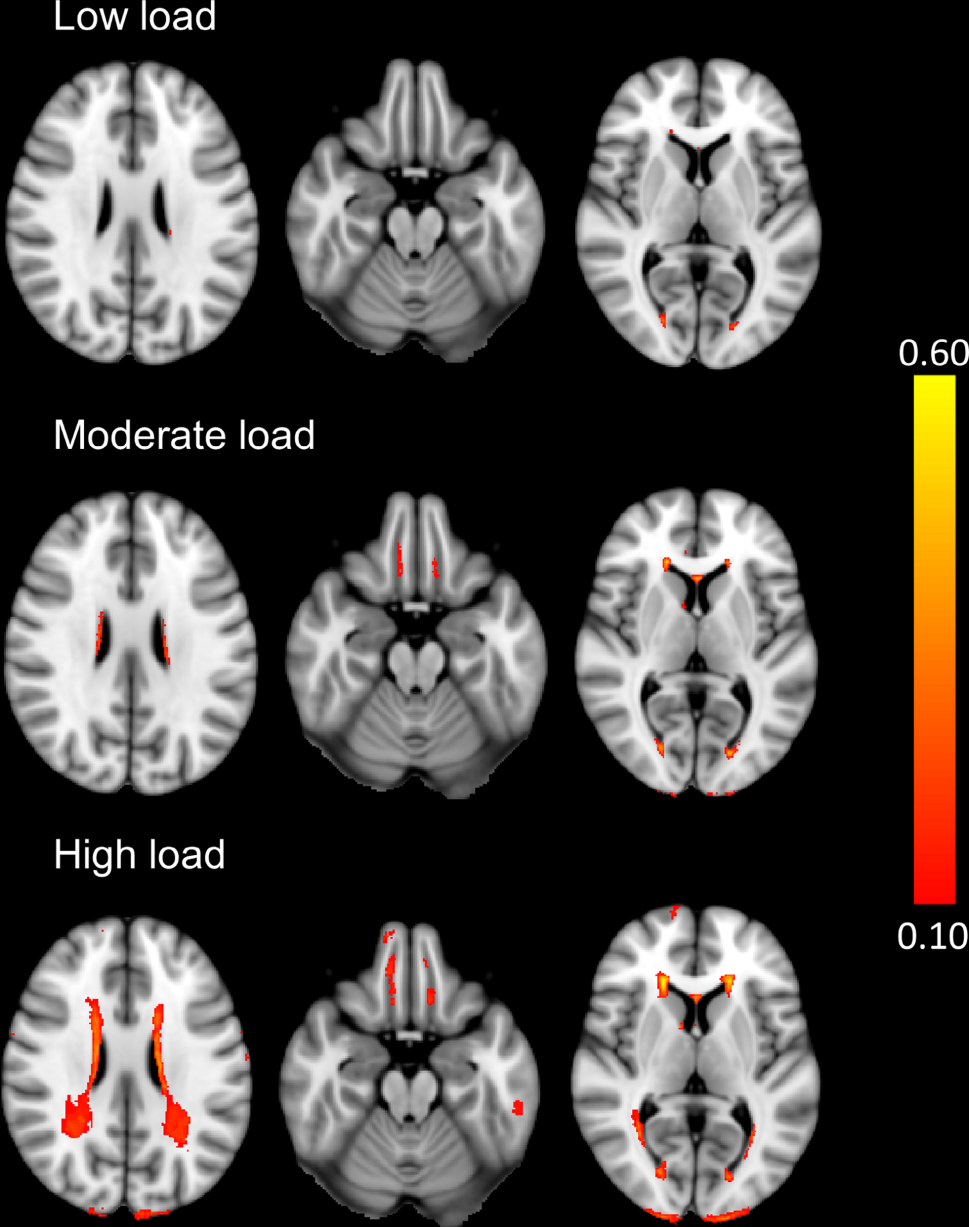
Group frequency maps showing the WMH distributions for participants categorized as having low, moderate, and high WM lesion loads.

Cortical reconstruction and volumetric segmentation of the T1‐weighted images was done with the FreeSurfer image analysis suite version 6.0 to obtain estimated total intracranial volume (eTIV).^74^ WMH were detected using the Lesion Segmentation Toolbox (LST) version 3.0.0, a MATLAB tool, implemented in SPM12 version 2.^75^, ^76^ This automated tool has been found to reliably and efficiently identify WMH across a range of neurologic conditions.^76^, ^77^, ^78^, ^79^ Bias‐corrected FLAIR images were first coregistered to normalized T1‐weighted anatomical images, which were segmented into cerebrospinal fluid, gray matter, and WM maps using SPM12 (http://www.fil.ion.ucl.ac.uk/spm/software/spm12). This information was combined with the coregistered FLAIR image to provide a lesion belief map for each class of tissue using the automated lesion growth algorithm (LGA) of LST. These maps were thresholded with a lesion probability threshold (κ) to generate an initial binary lesion map that was subsequently grown along voxels that appear hyperintense on the FLAIR image. To define the optimal threshold, T1 and FLAIR images from 11 randomly selected participants were manually segmented by a neuroradiologist, blinded to COC status, to establish ground truth lesion masks. A range of lesion volumes (1.34–24.3 mL; M = 6.51, SD = 8.33) was included in the training set. These same cases were then segmented at κ = 0.10, κ = 0.20, and κ = 0.30 using the LGA algorithm. Comparing the dice similarity coefficients (DSC) between the manually segmented images and the LGA‐generated lesion belief maps, it was determined that a κ = 0.10 was the optimal threshold (average DSC = 0.32; 100% specificity, 29% sensitivity). This was confirmed via visual inspection of the segmentation results. The DSC values are in line with other studies with overall low lesion loads, as errors in the segmentation have a greater impact when the lesion load is low.^80^, ^81^, ^82^ The remainder of the FLAIR data were then processed with LGA to derive whole brain WMH volumes for each participant. The WMH volume in milliliters (mL) was normalized to the eTIV to account for inter‐individual variations in brain size.^83^ These values were log10 transformed for analyses. As suggested by prior studies,^84^, ^85^ lesion load was categorized based on total WMH volume: minimal (<1 mL), low (≥1 through <5 mL), moderate (5–15 mL), and high (>15 mL). Figure 1 shows group frequency maps for the low, moderate, and high WMH lesion load classifications.

### Peripheral inflammatory biomarkers

Non‐fasting blood samples were collected by peripheral venipuncture immediately following the MRI or on a subsequent day. Plasma and serum were purified using standard procedures, and then, supernatant was pipetted into aliquots and stored at −80°C until time of assay. A selected set of 10 biomarkers representing various components of the inflammatory cascade were measured using commercially available enzyme‐linked immunosorbent assay (ELISA) kits. All analytes were measured in duplicate following manufacturers' instructions. Serum samples were assayed on the Luminex MagPix instrument using commercially available multiplex kits from EMD Millipore (Burlington, MA) to measure concentrations of IL‐4, IL‐6, IL‐8, IL‐13, and TNFα (Milliplex® Human High Sensitivity T Cell) and MPO (Milliplex® Human CVD Magnetic Bead Panel 2). Single ELISA assays were used for the following analytes: MCPI/CCL2 (serum; Human Uncoated; Thermo Fisher Scientific, Waltham, MA, USA), IP‐10/CXCL10 (plasma; LEGENDplex™; Biolegend, San Diego, CA, USA), sCD14 (plasma; Quantikine®, R&D Systems, Minneapolis, MN, USA), and sCD163 (plasma; Quantikine®; R&D Systems). Laboratory personnel were blinded as to the demographic and COC status of the participants. Four people were missing blood data, and one person was excluded because values were >3 SD above the mean on >3 biomarkers. The values for the cytokines/chemokines were log10 transformed to improve normality.

### Data analysis

Descriptive statistics were used to characterize the sample on demographic, HIV disease, CVD risk characteristics, and inflammatory biomarkers. We used two‐tailed independent samples *t*‐tests, Mann–Whitney *U*‐tests, chi‐squared tests, and Fisher's exact tests, as appropriate, to compare COC+ and COC− participants. A hierarchical multiple regression model was conducted to identify the independent association of cocaine use on WMH, after accounting for common risk factors. Variables were entered in a series of blocks in the following order: demographics (age in years, biological sex, race), clinical factors (CVD risk score, nadir and current CD4^+^ count, years since HIV diagnosis), and substance use (alcohol, marijuana, and cocaine; yes/no). A partial correlation was used to examine the strength of the relationship between WMH load and global cognitive function (*T*‐score) controlling for age. Finally, a series of analysis of covariance (ancova) models examined the association of WMH burden to each biomarker. Lesion load was the between‐subjects factor defined as minimal, low, moderate, or high, controlling or age. All analyses were conducted in SPSS 28.0.

## Results

### Sample characteristics

The sample included 32 COC+ and 78 COC− participants (Table 1). They were primarily male at birth (84%) and African American (72%) and had a mean age of 45.37 years (SD = 9.34). Compared to participants in the COC− group, COC+ participants were significantly older and less educated, and they were more likely to identify as African American. Participants had been diagnosed with HIV disease for a mean of 14.60 years (SD = 7.60), with no difference by COC status, but COC+ participants had significantly lower nadir and current CD4 T‐cell counts compared to COC− participants. Participants had been on ARV regimens for 12.33 years on average (SD = 6.82). Current ARV regimens were similar across the groups, with the majority (72%) being on an integrase strand transfer inhibitor‐based combination and 24% being on a regimen containing a protease inhibitor. The number of CVD risk factors ranged from 0 to 5, with a mean of 1.56 (SD = 1.15), and there was no difference by COC status. However, COC+ were more likely to currently smoke cigarettes, while COC− were more likely to have a BMI in the obese range.

**Table 1:** Sample characteristics by cocaine use status.

	COC+ (*N* = 32)	COC− (*N* = 78)	Statistic	*P*‐value
Demographic characteristics				
Male sex, *n* (%)	27 (84%)	62 (79%)	*Χ* ^2^ (1) = .35	0.554
Age in years, M (SD)	50.00 (8.58)	43.47 (9.01)	*t* (108) = 3.50	<0.001
African American race, *n* (%)	28 (88%)	51 (65%)	*Χ* ^2^ (1) = 5.48	0.019
Education in years, M (SD)	12.69 (1.96)	14.32 (2.60)	*t* (108) = −3.20	0.002
Global cognitive function, M (SD)	45.43 (6.66)	46.10 (6.49)	*t* (108) = −0.49	0.626
HIV disease characteristics				
Years since diagnosis	14.97 (7.13)	14.44 (7.83)	*t* (108) = 0.33	0.742
Nadir CD4+ T‐cell, Md (IQR)	76 (181)	238.5 (273)	*U* = 774.5	0.002
Current CD4^+^ T‐cell, Md (IQR)	460 (504)	814.5 (380)	*U* = 615	<0.001
Years on ART, M (SD)	11.81 (6.71)	12.54 (6.90)	*t* (107) = −0.50	0.616
Current ARV regimen, *n* (%)				
NRTI‐only	3 (9%)	5 (6%)	*Χ* ^2^ (3) = 0.38	0.945
NNRTI‐based	5 (16%)	14 (18%)		
INSTI‐based	23 (72%)	56 (72%)		
Other combination	1 (3%)	3 (4%)		
On a protease inhibitor, *n* (%)	10 (31%)	16 (21%)	*Χ* ^2^ (1) = 1.45	0.229
CVD risk factors				
Diabetes, *n* (%)	3 (9%)	9 (12%)	FET	1.000
Hyperlipidemia, *n* (%)	7 (22%)	23 (29%)	*Χ* ^2^ (1) = 0.66	0.416
Hypertension, *n* (%)	16 (50%)	24 (31%)	*Χ* ^2^ (1) = 3.63	0.057
Obesity, *n* (%)	5 (16%)	33 (42%)	*Χ* ^2^ (1) = 7.14	0.008
Cigarette smoking, *n* (%)	24 (75%)	24 (31%)	*Χ* ^2^ (1) = 18.05	<0.001
Prior CVD, *n* (%)	2 (6%)	2 (3%)	FET	0.578

INSTI, integrase strand transfer inhibitor; NNRTI, non‐nucleoside reverse transcriptase inhibitor; NRTI, nucleoside reverse transcriptase inhibitor.

Participants in the COC+ group had been using cocaine regularly for an average of 18.28 years (SD = 10.70). The majority (66%) reported smoking as their primary route of administration; all others reported nasal administration. In the 90 days prior to enrollment, they had used cocaine on an average of 23.75 days (SD = 20.95). While the overall prevalence of use was high for current alcohol (75%) and cannabis (48%), COC+ participants were significantly more likely than COC− participants to have used these substances (both *P* < 0.05).

### Predictors of WMH burden

**Figure 2:**
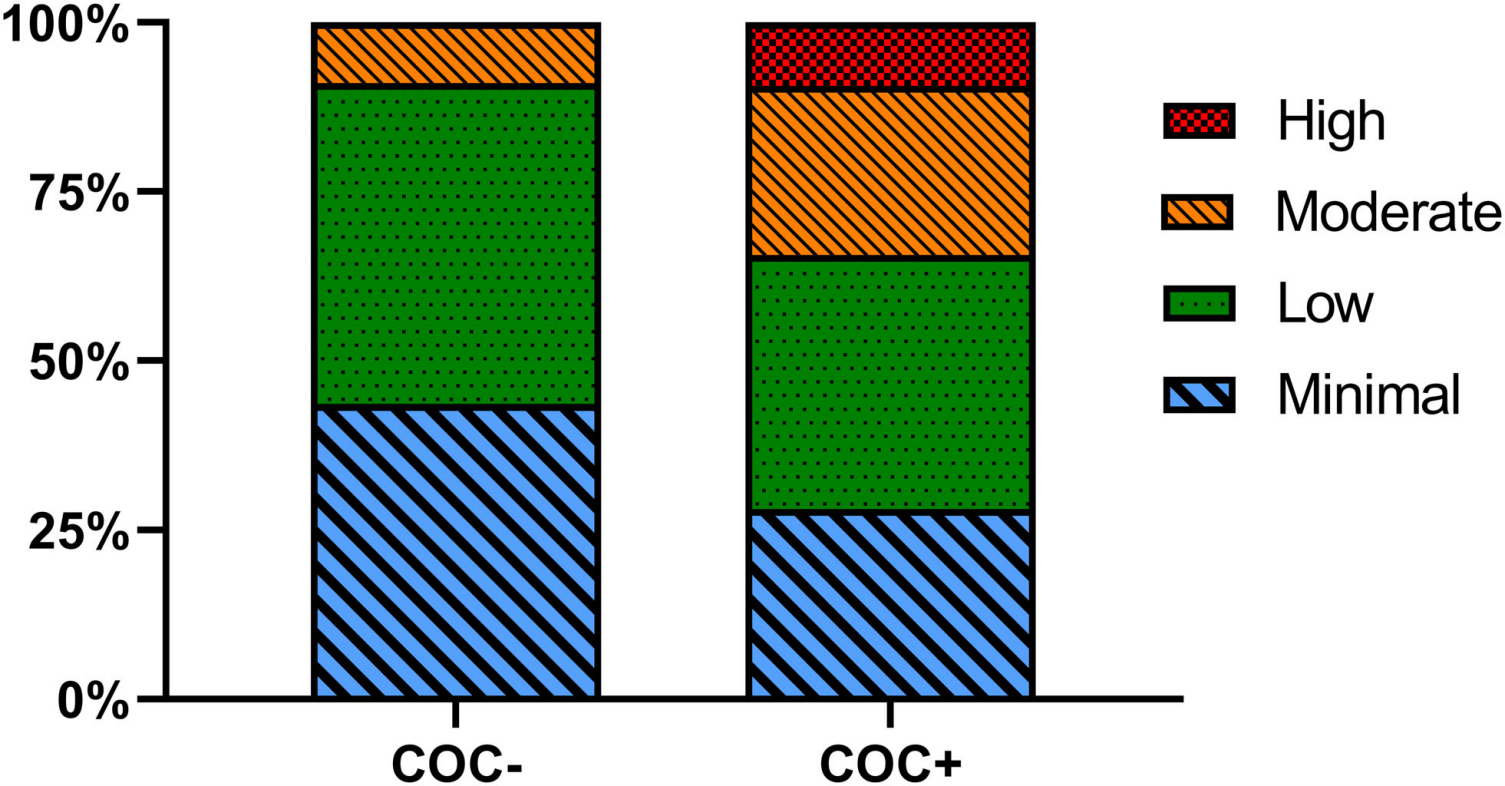
Stacked bar graphs show the proportion of participants in each COC group categorized by white matter burden.

The median WMH volume was higher for COC+ (Median = 1.9e‐6, IQR = 3.4e‐6; Min = 8.1e‐8, Max = 1.5e‐5,) than for COC− (Median = 7.8e‐7, IQR = 1.3e‐6, Min = 1.1e‐8, Max = 9.0e‐6) (*U* = 1695, *P* = 0.003). Figure 2 illustrates the proportion of participants in each group categorized by WMH burden (χ^2^(2) = 13.48, *P* = 0.004). Compared to the COC− group, COC+ participants were more likely to be categorized as high (9% vs. 0%) and moderate (25% vs. 9%) lesion burden and less likely to have minimal lesion burden (28% vs. 44%). Table 2 summarizes the results of the hierarchical regression model predicting WMH volume. The final regression model was statistically significant [(*F* (10, 99) = 3.44, *P* < 0.001, *R* ^2^ = 0.26). Step 1 included demographic factors, and age was associated with higher WMH volume. Step 2 added clinical factors. As expected, CVD risk score was associated with higher WMH volume. None of the HIV clinical variables were predictive. Step 3 added the substance use factors, and cocaine was associated with higher WMH volume. In the final model, age, CVD risk, and cocaine use were independent predictors of WMH burden.

**Table 2:** Hierarchical linear regression model predicting WM lesion burden.

	Step 1	Step 2	Step 3
	Demographics (β)	Clinical factors (β)	Substance use (β)
Age in years	0.37***	0.30*	0.17
Male sex	0.02	0.04	0.09
African American race	0.11	0.03	−0.04
CVD risk score		0.29**	0.29**
Nadir CD4 T‐cell count		−0.03	−0.05
Current CD4 T‐cell count		−0.02	0.06
Years since HIV diagnosis		−0.09	−0.05
Alcohol use			−0.08
Marijuana use			−.12
Cocaine use			.28*
Block	*F* = 5.66** *R* ^2^ = 0.14	*F* = 3.85*** *R* ^2^ = 0.21	*F* = 3.44*** *R* ^2^ = 0.26

β, standardized coefficients; *R* ^2^, proportion of explained variance in WM lesion burden.

* *P* < 0.05.

** *P* < 0.01.

*** *P* < 0.001.

### Relationship between WMH and cognitive function

**Figure 3:**
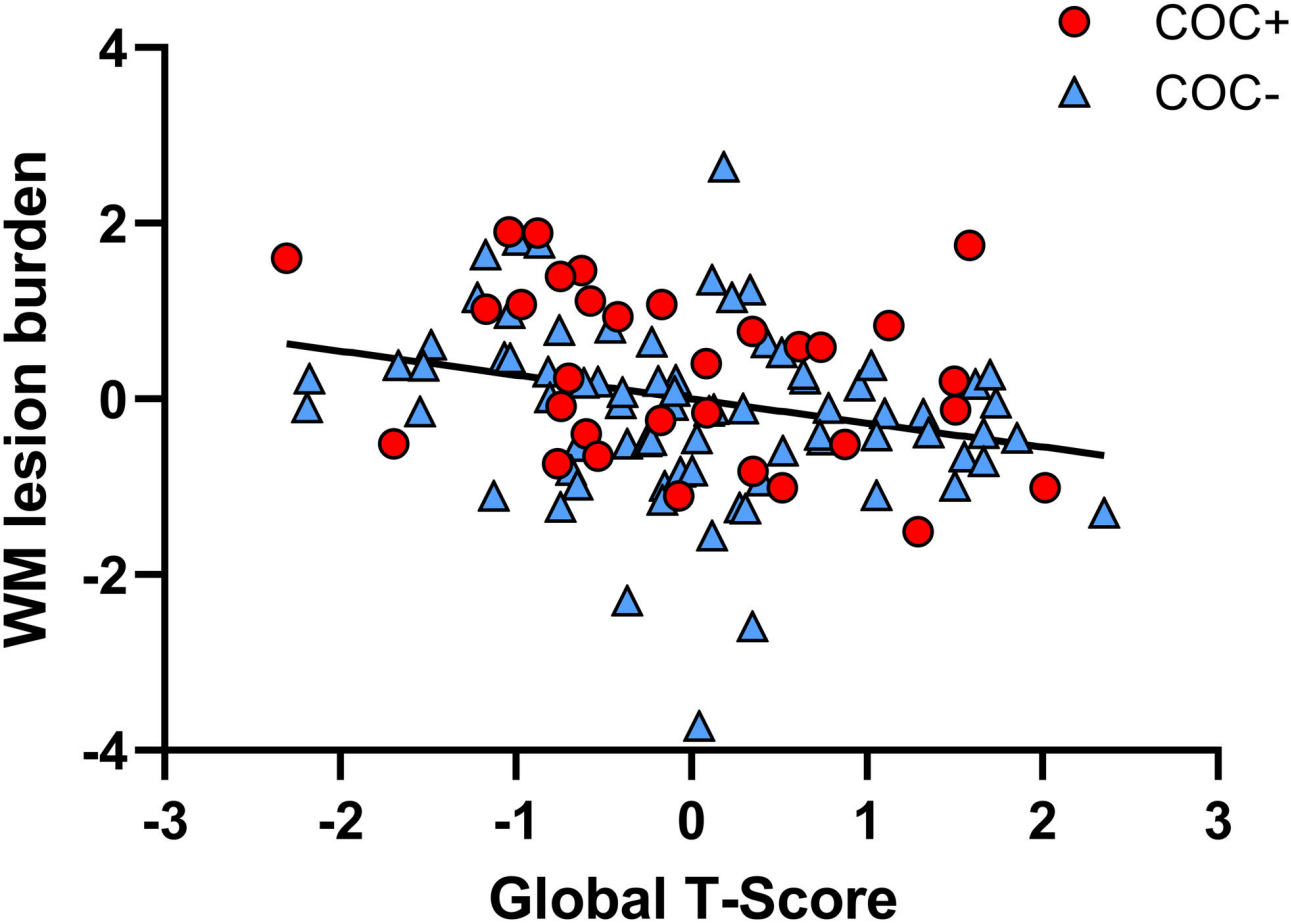
The scatterplot shows the negative correlation between WM lesion burden and global *T*‐score across the full sample. The standardized residuals from the partial correlation controlling for age are plotted. The individual data points are coded by COC status to illustrate that the relationship was similar across the groups.

WMH volume was negatively correlated with global *T*‐score (*r*_partial_ = −0.27, *P* = 0.004), suggesting that greater WMH burden is associated with poorer cognitive function (Fig. 3). The strength of the correlation was similar for the COC+ (*r*_partial_ = −0.338, *P* = 0.063) and COC− (*r*_partial_ = −.244, *P* = 0.032) groups. In an exploratory analysis, we examined the correlation with domain *T*‐scores. WMH volume was negatively associated with executive function (*r* = −0.19, *P* = 0.049), learning (*r* = −0.29, *P* = 0.002), and memory (*r* = −0.33, *P* < 0.001).

### Exploratory correlations of inflammatory biomarkers and WMH burden

After accounting for age, the COC group had comparable levels for all of the inflammatory markers except IL‐13 (Table 3). Table 4 summarizes the results of the ancova models comparing biomarker values across WMH categories. Controlling for age, concentrations of IP‐10 and MPO were significantly different across the four categorizations. Specifically, participants who were categorized as having moderate or high lesion burden had the highest concentrations of IP‐10 and the lowest concentration of MPO. However, the group differences did not pass family wise error correction for multiple comparisons. Since IP‐10 and MPO did not differ by COC status, we did not pursue mediation analysis.

**Table 3:** Biomarkers by cocaine use status, controlling for age.

Peripheral biomarkers	Estimated marginal means, M (SE)		ancova models	
	COC+ (*N* = 32)	COC− (*N* = 78)	*F*‐value	*P*‐value
sCD163, ng/mL	442.96 (39.20)	444.51 (25.44)	<0.01	0.974
sCD14, μg/mL	1.38 (0.08)	1.33 (0.05)	0.27	0.607
TNF‐α, pg/mL	0.95 (0.03)	0.93 (0.02)	0.41	0.525
IL‐6, pg/mL	0.09 (0.09)	0.26 (0.06)	2.52	0.116
IL‐4, pg/mL	0.68 (0.10)	0.87 (0.06)	2.43	0.122
IL‐13, pg/mL	0.07 (0.12)	0.38 (0.08)	4.46	0.037
IL‐8, pg/mL	1.01 (0.05)	1.04 (0.03)	0.37	0.546
IP‐10, pg/mL	0.92 (0.13)	0.90 (0.09)	0.03	0.874
MCP1, pg/mL	2.01 (0.07)	2.05 (0.05)	0.24	0.625
MPO, μg/mL	1.00 (0.12)	0.91 (0.08)	0.29	0.589

**Table 4:** Biomarker values and association to WMH categorization.

Peripheral biomarkers	Estimated marginal means, M (SE)				ancova models	
	Minimal *N* = 41	Low *N* = 48	Moderate *N* = 16	High *N* = 3	*F*‐value	*P*‐value
sCD163, ng/mL	419.14 (34.19)	426.84 (30.59)	560.22 (59.32)	555.91 (124.41)	1.76	0.160
sCD14, μg/mL	1.34 (0.07)	1.37 (0.06)	1.26 (0.12)	1.47 (0.25)	0.35	0.792
TNF‐α, pg/mL	0.93 (0.03)	0.92 (0.03)	0.98 (0.05)	0.98 (0.10)	0.45	0.721
IL‐6, pg/mL	0.24 (0.08)	0.17 (0.07)	0.15 (0.13)	0.55 (0.28)	0.72	0.543
IL‐4, pg/mL	0.75 (0.09)	0.82 (0.08)	0.85 (0.15)	1.31 (0.32)	0.95	0.418
IL‐13, pg/mL	0.28 (0.11)	0.28 (0.10)	0.25 (0.19)	0.65 (0.41)	0.29	0.829
IL‐8, pg/mL	1.05 (0.04)	0.98 (0.04)	1.14 (0.08)	1.25 (0.16)	2.00	0.119
IP‐10, pg/mL	0.79 (0.11)	0.86 (0.10)	1.18 (0.20)	1.97 (0.41)	3.13	0.029
MCP1, pg/mL	1.95 (0.06)	2.08 (0.06)	2.12 (0.11)	2.24 (0.24)	1.03	0.385
MPO, μg/mL	1.15 (0.11)	0.85 (0.10)	0.80 (0.18)	0.11 (0.39)	3.02	0.033

## Discussion

This study investigated the contribution of ongoing cocaine use to WMH in persons with treated HIV disease who had sustained viral suppression. Our principal finding is that cocaine use in PWH was associated with greater WM lesion burden, even after accounting for age and CVD risk factors. Moreover, higher WM lesion burden was correlated with poorer cognitive function, underscoring the role of vascular processes in the development of NCI in PWH independent of COC status. While chronic HIV disease may contribute to the development of cerebral small vessel disease, it appears that age, CVD risk, and cocaine are robust predictors of WMH with high relevance for PWH. Finally, circulating neurotoxic chemokines linked to endothelial functioning were associated with WM lesion burden, suggesting a potential role of inflammatory processes in the pathogenesis of cerebrovascular insults in the context of chronic HIV infection.

This study was specifically designed to test the effects of cocaine on health outcomes in PWH, comparing individuals with ongoing cocaine use to those with no history of substantive cocaine use. Our results support the independent role of ongoing cocaine use on burden of WMH. A recent study did not find an association between cocaine use and WMH in PWH, but groups were defined by a past history of cocaine use disorder rather than current use.^20^ Acute cocaine administration inhibits catecholamine reuptake at sympathetic nerve terminals, with potent vasoconstrictive effects.^86^ This persistent hypoperfusion can result in ischemic damage to WM.^87^, ^88^ Future research is needed to empirically test whether recovery from cocaine use disorder mitigates or reverses the effects of past cocaine use on WMH burden.

Consistent with the extensive literature on cerebral small vessel disease,^39^, ^89^ CVD risk was strongly associated with WMH. Interestingly, cocaine use was unrelated to overall CVD risk score, suggesting that CVD risk did not mediate the relationship between cocaine and WMH burden. COC+ participants were more likely to smoke cigarettes but also less likely to have a BMI in the obese range, balancing out overall risk score. However, there was a trend for COC+ participants to have a higher prevalence of hypertension. While the link between chronic cocaine use and CVD risk is well established,^90^, ^91^ several factors likely contributed to our results. First, the prevalence of CVD risk factors was high overall, as HIV disease is associated with increased risk.^92^, ^93^ Second, our scale did not account for severity of each risk factor (e.g., controlled versus uncontrolled disease). Third, a history of a cerebrovascular events, such as stroke, was an exclusion criterion for the study, and persons who used cocaine were more likely to be excluded for this reason. While our exclusions may limit the generalizability of findings with respect to prevalence of CVD risk, it allowed us to better isolate the effects of cocaine on WMH.

Despite prior reports that HIV disease confers an elevated risk of WMH,^19^, ^20^, ^21^, ^22^, ^23^ the pathophysiology remains uncertain. Consistent with other studies,^20^, ^23^, ^46^, ^47^ we found no relationship between WMH and HIV disease characteristics, such as time since diagnosis and CD4 counts of immune function. Given our inclusion criteria, study participants were fairly homogeneous in terms of their HIV disease; all had a diagnosis for >5 years and/or a low nadir CD4 suggestive of long‐standing disease and were on combination ARV with viral suppression for >1 year. COC+ participants did have a history of more severe immunosuppression and less robust immune reconstitution, but these differences did not account for the effects of cocaine on WMH.

At the cellular level, cerebral small vessel disease is believed to result from endothelial dysfunction and subsequent exposure of the arterial vessel wall, making it susceptible to thrombosis.^35^, ^94^ Although our analyses were exploratory, we found that concentrations of IP‐10, a chemokine implicated in the induction of leukocyte migration to the site of inflammation,^95^, ^96^ were associated with higher WMH burden. IP‐10 is believed to inhibit endothelial healing,^97^ and it has been suggested as a marker of thrombosis.^98^, ^99^ We also found that MPO, an inflammatory enzyme, was lower in participants with higher WMH burden. MPO is expressed following acute neuronal injury, triggering the production of the bactericide hypochlorous acid, a strong oxidant that can cause local tissue damage and amplify the inflammatory cascade.^100^, ^101^ Elevated MPO levels are associated with inflammation and oxidative stress and are predictive of CVD risk, including thrombosis and stroke.^102^ One study of stroke‐free adults found that elevated MPO was associated with greater burden of WMH.^103^ However, an analysis of >1700 adults enrolled in the Framingham Heart Study found that WMH and silent cerebral infarcts were associated with lower MPO while cerebral microbleed was associated with higher MPO.^38^ The authors suggested that different inflammatory pathways may be involved in the pathogenesis of ischemic versus hemorrhagic MRI markers. The mechanism underlying the lower levels of circulating MPO in persons with higher WMH is uncertain but is consistent with the hypothesis that MPO deficiency, via a diminished antioxidant response, may promote ischemic neuronal injury.^104^, ^105^ Since there were no differences in the levels of these inflammatory biomarkers, the potential mechanisms do not appear to mediate the effects of cocaine on WMH.

Strengths of this study include our use of comprehensive assessments to characterize the sample and rigorous eligibility criteria related to both substance use and HIV disease to minimize confounds and maximize power. Our study also has some limitations. First, we did not include an HIV‐negative comparison group because the goal of the larger project was to identify neural correlates of NCI in PWH. Second, the sample size of 110 is relatively modest, but the study was designed specifically to isolate the effects of cocaine on HIV‐related outcomes. With clearly delineated groups, our power to detect differences attributed to cocaine use was strengthened. Third, we used a research‐domain automated pipeline for lesion segmentation, as manual delineation of WM lesions is labor intensive with considerable inter‐ and intra‐rater variability.^106^ While the LGA method has been found to be valid and robust,^107^, ^108^, ^109^ our own data suggested high specificity but low sensitivity, so we may have underestimated WMH burden in our sample. Furthermore, the lesion segmentation toolbox we used did not quantify the location of the WMH. Visual inspection of the FLAIR images revealed that most large lesions were periventricular, consistent with prior studies in HIV disease,^20^ but deep WMH were also common although generally smaller. Recent studies suggest that sub‐classifying WMH according to location and severity may reveal more specific information about NCI.^110^ Fourth, our study did not assess all possible relevant outcomes and constructs that may contribute to WMH burden. For example, we only examined WMH, rather than the full‐spectrum of cerebral small vessel disease. While our analysis did evaluate some social determinants of health outcomes, there are likely a host of other important socioeconomic factors that our analysis did not consider, such as food insecurity and poverty. Finally, our cross‐sectional analysis does not allow for causal inferences, and we cannot exclude the possibility of residual confounding.

Our principal finding is that PWH who use cocaine have higher WM lesion burden, even after accounting for CVD risk factors, that contribute to cognitive impairment. While the relative burden of WMH remained modest for most participants, these lesions represent only the end of a continuous spectrum of WM injury and neurodegeneration.^111^, ^112^ As the sample was relatively young, with a mean age of 45, these WMH may portend future cerebrovascular events, vascular dementia, and frailty as PWH age. Clinically, as we observed, even small lesion load can predict cognitive impairment in middle‐age cognitively healthy adults.^113^ Longitudinal studies can help identify patients with progressive changes in brain structure and integrity, and ultimately determine the clinical significance of WMH observed in PWH. In sum, given the deleterious role of cocaine use in promoting neural injury and NCI in PWH, our results reinforce the importance of substance use screening for all patients receiving HIV care and referral to treatment and support services when indicated to minimize additional WM injury that may exacerbate NCI.

## Author Contributions

Conception and design of the study: CSM, RPB, and SLT. Acquisition, analysis, or interpretation of data: CSM, RPB, and CDL. Statistical analysis: CSM, RPB, and SLT. Drafting of the manuscript or figures: CSM, RPB, and SLT. Critical revision of the manuscript: CSM, RPB, SLT, CDL, KA, and MJG. Obtaining of funding: CSM and SLT.

## Conflict of Interest

The authors declare that the research was conducted in the absence of any commercial or financial relationships that could be construed as a potential conflict of interest.
